# Effect of COVID-19-Related Home Confinement on Sleep Quality, Screen Time and Physical Activity in Tunisian Boys and Girls: A Survey

**DOI:** 10.3390/ijerph18063065

**Published:** 2021-03-16

**Authors:** Rihab Abid, Achraf Ammar, Rami Maaloul, Nizar Souissi, Omar Hammouda

**Affiliations:** 1 Research Unit: Physical Activity, Sport, and Health, UR18JS01, National Observatory of Sport, Tunis 1003, Tunisia; isseps.rihababid@gmail.com (R.A.); n_souissi@yahoo.fr (N.S.); 2 Institute of Sport Science, Otto-von-Guericke University, 39106 Magdeburg, Germany; 3 Interdisciplinary Laboratory in Neurosciences, Physiology and Psychology: Physical Activity, Health and Learning (LINP2), UPL, Paris Nanterre University, UFR STAPS, 92000 Nanterre, France; hammouda.o@parisnanterre.fr; 4 Research Laboratory, Molecular Bases of Human Pathology, LR19ES13, Faculty of Medicine, University of Sfax, Sfax 3029, Tunisia; rami.maaloulll@gmail.com

**Keywords:** COVID-19, confinement, sleep quality, screen time, physical activity, children

## Abstract

COVID-19 home confinement has led to a stressful situation for children around the world and affected their lifestyle. The present study aimed to investigate the effect of these restrictions on sleep quality, screen time (ST) and physical activity (PA) in Tunisian children with a special focus on gender differences. An online survey was launched in April 2020. Questions were presented in a differential format, with expected responses related to “before” and “during” confinement. Participants (52 boys and 48 girls, age: 8.66 ± 3.3 years) responded to the Pittsburgh Sleep Quality Index (PSQI), the digital media use, and the Ricci and Gagnon sedentary behavior questionnaires. Findings revealed that COVID19 home confinement had a negative effect on all the considered parameters (*p* < 0.05). Significant effects of gender were found on sleep disturbances (*p* = 0.016, np^2^ = 0.05), subjective sleep quality (*p* < 0.01, np^2^ = 0.07), global score of PSQI (*p* = 0.01, np^2^ = 0.01) and nocturnal and global screen time (*p* < 0.001, np^2^ = 0.09) with poorer sleep and higher screen time in girls compared to boys during home confinement. A significant correlation was shown between Global ST and PSQI score (r = 0.39, *p* < 0.001). Programs of PA for children and sensitization campaigns against the use of screens have been deemed urgent with special focus oriented to girls.

## 1. Introduction

In December 2019, a novel outbreak of pneumonia was identified in Wuhan (Hubei, China) and named as coronavirus disease 19 (COVID-19) by the World Health Organization [1]. Due to the fast spread of this abrupt virus, infections were no longer limited to China, but included Thailand, Japan, Korea, the USA, Spain, Italy and France in early 2020 [2]. Spread around the world, it was soon considered as a public health emergency of international concern and announced as a universal pandemic [1]. On 2 March 2020, the Tunisian ministry of health announced the first case of COVID-19. On 20 March 2020, Tunisian president announced a total confinement of two months indicating that leaving home was only permitted in extreme cases. Schools, colleges and higher schools also kept their doors locked. On the one hand, private schools have put in place process to ensure the continuity of courses, most often through online courses sessions. On the other hand, an educational channel has broadcasted courses recorded by teachers.

Prolonged school closing, combined with home confinement, can have negative effects on children’s physical and psychological health. When children are out of school, they have much longer screen time (ST), irregular sleep patterns and less physical activity (PA) [3]. These modifications may not only increase daytime stress, anxiety and depression levels but also disrupt sleep [4].

Media companies have recently indicated a huge increase in social media use [5]. People confined to their home watch TV and check social media more often, which explains the recently reported huge increases in gaming and total TV usage (+60% in USA) and social media usage during COVID-19 home confinement [6,7]. Previous reports indicate that frequent use of social media platforms has a negative effect on mental health, circadian rhythms and sleep outcomes [8]. In the context of COVID-19, a recent online survey was conducted to comprehend the effect of lockdown on screen exposure time (ST) and sleep behavior on Indian school children [9]. Results demonstrated a huge increase of ST. Similarly, sleep inertia and sleep latency were higher during the lockdown.

Stability of the circadian sleep–wake rhythms is important for optimal childhood development [10]. Gender effect on sleep quality remains less well described essentially in childhood. Previous researches reported that girls recorded longer time spent in bed with an earlier bedtime and later wake time than boys [11]. However, other studies found no gender differences on sleep latency [12], sleep quality and sleep perturbation [13].

Any perturbation in duration or quality of sleep is considered as sleep disturbance and is related to poorer physical and psychosocial functioning [14] and may lead to overweight, obesity and behavioral problems [15]. Similarly, exposure to artificial light at night (ALAN) is often linked to adverse health effects. Indeed, a high rate of insomnia [16], a poorer sleep quality and a higher rate of fatigue [17] as well as a delayed sleep phase disorder [18] were previously identified in individuals exposed to intense nocturnal artificial light by suppressing melatonin [19]. Sleep quality is most affected by digital media consumption that occurs within the two hours prior to bed time [20]. As the melatonin is more sensitive to light in children compared to adults (i.e., double suppression rate at night) [21], children seem to be more susceptible to the negative health effects of ALAN specifically during the COVID-19 home confinement context.

On the other hand, physical activity (PA) has beneficial effects on mental health in children and adolescents [22]. Girls however seem less active than boys. Only 11% of girls achieve the recommended 60 min or more of activity per day compared to 25% of boys [23]. During PA, the brain is activated by increasing blood flow to essential areas that may stimulate learning [24]. Also, PA is associated with better sleep quality [25]. Recent research reported that inadequate sleep may contribute to reduced PA levels, thus emphasizing the strong bidirectional relationship between PA and sleep [26]. In this way, exercise has long been considered as a non-pharmacological treatment option for sleep disturbances [27].

Therefore, the aims of the present study were to investigate the effect of COVID-19 home confinement on sleep quality, digital media use and the level of PAfor Tunisian boys and girls.

## 2. Materials and Methods

### 2.1. Sample, Recruitment and Inclusion Criteria

The data hereby presented were gathered through a cross-sectional online survey starting 24 April 2020, and was collected until 10 May 2020, when containment measures had already eased. School-aged children (5–12 years old) were randomly selected from different socioeconomic classes. Employment status and level of education of parents are presented in Table 1.

Participants completed an anonymous online survey, after reading and signing with their parents the written consent form. The assistance of one of the parents for the questionnaire responses was deemed mandatory to maintain the credibility and accuracy of the answers [28]. The study was conducted according to the Declaration of Helsinki and approved by the Local Institutional Review Board.

### 2.2. Sample Size

The sample size was calculated a priori, using the software G*Power. Given the pioneer character of this study, the effect sizes were estimated to 0.5 (medium effect). A sample size of 100 participants was therefore enough to reach a desired statistical power of 0.95 at α = 0.05.

### 2.3. Survey Questionnaires

The survey included fifty questions on sleep, PA and ST. The questions were presented in two formats: “before” and “during” confinement. It contains an introductory page to describe the background and the goals of the survey. The answers of participants were anonymous and confidential and are only for research purposes. The survey was designed by scientists and academics. It was uploaded and shared on the Google online survey platform (Google Forms). A link to the electronic survey was distributed via e-mails and social media.

### 2.4. Sleep Quality

Sleep quality was assessed using the global composite score of the Pittsburg Sleep Quality Index (PSQI) [29]. This score evaluates subjective sleep quality during the previous month. It consists of 19 self-rated questions. These items are grouped into scores with the following seven parameters: sleep quality, sleep latency, sleep duration, sleep efficiency, sleep disturbances, use of sleep medication and daytime dysfunction. Each component is weighted equally on a 0–3 scale. The total score for the PSQI varied from “0” to “21”, where “0” indicates no trouble and “21” indicates severe problems in all areas. APSQI total score>5is indicative of poor sleep. A global score greater than 5 yielded a diagnostic sensitivity of 89.6% and specificity of 86.5% (kappa = 0.75, *p* < 0.001), with an internal consistency *α* = 0.83 and test–retest reliability, *r* = 0.85, in distinguishing “good” and “poor sleepers” [30].

### 2.5. Digital Media Use

The use of digital media was assessed by asking children for the frequency of digital media use, time of media use and the frequency of the utilization during the day and in the last 2 h before bedtime during the previous month. They were asked about preferred time for using digital devices (during the day or at night), the number of times they had used digital devices at night per one week the previous month. Answers included one of 8 selections: “every day, 6 times per week, 5 times, 4 times, 3 times, 2 times, 1 time, I don’t use digital devices at night”. The duration of usage per one time in minutes: “How long do you use it per once?”. We selected five types of electronic media based on SergeTisseron’s questionnaire which is intended for children in elementary and middle schools [31]. It includes the measurement of TV, tablet, computer, mobile phone and internet usage. To calculate daytime and nighttime screen exposure, we added up durations of usage of all instruments.

### 2.6. Level of Physical Activity

To assess the level of PA of the participants, we administered the Ricci and Gagnon sedentary behavior questionnaire (RG; Montreal University, modified by Laureyns and Séné) [32]. The score of the questionnaire was used to classify participants as sedentary, moderately active or very active (sedentary <18; moderately active between 18 and 35 and very active >35). It contains 3 parts: sedentary behavior, leisure PA and daily PA. Daily PA evaluates the duration and intensity of common daily activities such as housework, gardening, walking, and climbing stairs. While the leisure PA part evaluates sports and recreational activities.

### 2.7. Statistical Analyses

All data were analyzed using SPSS statistical software (v.23, IBM, New York, NY, USA). Results were presented as Mean ± standard deviation (SD) in tables and text. The normality of the distribution was verified with the Shapiro– Wilk W-test and Levene’s test was also performed to determine the homogeneity of variance. Normality and homogeneity were assumed for *p* > 0.05. When normality was verified, a parametric test (Bonferroni) was performed.

The effects of confinement and gender were assessed by two-way analysis of variance (ANOVA) with repeated measures [confinement (before × during) and gender (boys × girls)]. When a significant difference was detected between genders, a Bonferroni posthoc test or Wilcoxon test was performed for pair-wise comparison. Effect sizes (ES) were calculated as partial eta-square (η_P_^2^) and interpreted as trivial η_P_^2^ < 0.2, small 0.2 ≤ η_P_^2^ < 0.5, moderate 0.5 ≤ η_P_^2^ < 0.8 and large η_P_^2^ ≥ 0.8 [33]. To evaluate the magnitude of evolution (before to during confinement) of a variable’s value, we calculated the % of change as Mean ± SD. All values of “before” and “during” confinement are made in Means ± SD format.

## 3. Results

### 3.1. Sample Description

Total responses received were 113, of which 100 participants (52 boys and 48 girls, mean age: 8.66± 3.3 years) within the 5–12years of age range were selected for the study.

### 3.2. Effect of Home Confinement on Sleep Behavior

Reponses to the different PSQI questions in respect to before and during home confinement are presented with respect to Table 2.

Statistical analyses showed a significant main effect of “Confinement” on the majority of the PSQI items (*p* < 0.001). Particularly, in both genders the scores of subjective sleep quality, sleep latency, sleep disturbances and daytime dysfunction significantly increased (*p* < 0.001) associated to poorer sleep. Also, habitual sleep efficiency significantly decreased from “before” to “during” home confinement (*p* < 0.001). However, there were no significant changes in sleep duration. Furthermore, the two-way ANOVA showed a significant main effect of gender on subjective sleep quality (*p* = 0.01) and sleep disturbances (*p* = 0.016) with higher values in girls compared to boys during home confinement (*p* < 0.001).

Statistical analysis showed a significant main effect of “confinement” (*p* < 0.001; ηp^2^ = 0. 76) on total PSQI score with higher score “during” compared to “before” confinement in all population and for girls and boys (Δ% = 82.26, Δ% = 175.19%, and 173.44%, respectively). A significant main effect of “gender” was registered on global score of PSQI (*p* = 0.01 and ηp^2^ = 0.07) with higher values in girls compared to boys “during” home confinement (*p* = 0.01).

### 3.3. Effect of Home Confinement on Screen Time

Responses to daily ST recorded before and during home confinement are presented in Table 3.

Statistical analyses showed a significant main effect of “Confinement” for all population on both diurnal (+150%) and nocturnal exposition (+93.33%) to digital devices (*p* < 0.001). Particularly, in both genders the diurnal ST (Δ% = 164.13% for boys and 148.8% for girls) as well as the nocturnal ST (Δ% = 8.21% for boys and 7.14% for girls) increased from “before” to “during” home confinement (*p* < 0.001). Furthermore, the Two-way ANOVA showed a significant main effect of gender on diurnal (*p* = 0.013) and nocturnal (*p* < 0.001) exposition to digital devices with higher values in girls compared to boys at during home confinement (*p* = 0.013 for diurnal and *p* = 0.004 for nocturnal screen time).

Statistical analysis showed a significant main effect of “confinement” on global ST score with higher score at during compared to before confinement in both genders (Δ% = 176.37% for girls and 189.13% for boys). A significant main effect of “gender” was registered on global exposition (*p* < 0.001, ηp^2^ = 0.1) with higher values in girls compared to boys at during home confinement (*p* = 0.001).

### 3.4. Effect of Home Confinement on Physical Activity

Reponses to level of PA before and during confinement are presented in Table 4.

Statistical analyses showed a significant main effect of “Confinement” on all items of the PA questionnaire (*p* < 0.001) as well as on the total PA score (*p* < 0.001). Particularly, in both genders’ leisure (Δ% ≃ −35%), daily (Δ% = −16% for boys and−27% for girls) and total PA (Δ% = −7% for boys and −17% for girls) decreased from “before” to “during” home confinement (*p* < 0.001). However, no significant effect of gender was recorded in any of the PA items.

More descriptive results (mean, median, maximum and minimum) are presented in the supplementary tables (Tables S1–S3).

## 4. Discussion

The present study was designed to examine, for the first time, the effect of home confinement on sleep quality, digital media use and PA in Tunisian boys and girls aged 5 to 12 years. The main findings showed that sleep quality was deteriorated during home confinement essentially for girls. Tunisian children decreased their PA levels and increased their ST during the confinement. These findings should be taken into consideration to promote healthy lifestyle strategies for children during and after confinement. During the confinement period, Tunisian children were negatively affected by disturbing their sleep and reporting higher PSQI scores (+82.22%) which incur poorer sleep. According to previous study, Cellini et al. [34] proved that a sleep–wake rhythm had changed for young people with going to bed and waking up later and spending more time in bed during lockdown. Notably, poor sleep quality was linked to higher levels of depression, anxiety, and stress, which are presumable effects of long periods of confinement [34]. The present findings showed also that boys had relatively better sleep quality than girls during confinement. These findings are in accordance with a recent study that approved the sex differences in human sleep behavior, polysomnographic and electroencephalographic measures mostly near puberty [35]. Precisely, this study showed more sleep problems and bad sleep quality in girls.

These gender differences have been suggested to be due to the influence of the hormonal changes which affect sleep architecture across the lifespan, including childhood [35]. The European Academy for Cognitive Behavioral Therapy for Insomnia [36] indicated that most individuals are exposed to a novel stressful situation of unknown duration because of being forced to stay at home. This situation may not only increase stress, anxiety and depression levels but also disrupt sleep. Effectively, during the lockdown sleep habits are challenged by various factors such as reducing exposure to sunlight, reducing PA and increasing psychological distress [37].

Otherwise, the present study showed an increase of ST (+111.11%) as well as diurnal (+150%) and nocturnal (+93.33) digital media use of Tunisian children during COVID-19 outbreak. These worrying results shed light on the dire consequences for Tunisian children’s health due to strict confinement [34]. The previous few studies published to examine the effect of lockdown on screen time are in line with our results [38,39]. Xiang et al. [38] reported a huge increase of 4.7 h per week of total screen leisure time in Chinese youths. Also in Italy, the frequency of digital media use during the last 2 h before going to bed had increased (i.e., from 27 activities a week before the restriction to 31 activities) under lockdown [34].

Particularly, the present findings showed a higher utilization of digital devices on girls. Several studies which investigated the gender-related differences in usage of screen-based technologies obtained contradictory results [40]. Screen time was higher in adolescent females [41]. In contrast, another study demonstrated that boys spent more screen time than girls [42]. Most of the previous studies have shown mixed results about the usage of screen-based technologies for any gender. Girls watched more TV and video games, while boys spent more time on computer and internet [43]. These findings inspire new research works to check different causes that influence children’s preferences.

Taken together, it can be argued that the increase of usage of the digital media at night had disturbed the sleep of the children in the present study. These findings were associated with many studies demonstrating that the exposure to ALAN is often linked to adverse health effects, such as disruption of the circadian phase and sleep deterioration [44]. In this sense, different types of electronic screen media have repeatedly been linked to impaired sleep [45]. Experts from the National Institute for Sleep and Vigilance admit that whatever type of new technologies: screens, tablets, computers and mobiles are disturbing our lifestyle, especially our sleep habits [44].

Otherwise, the results of the present study showed that children are more inactive during the lockdown. According to previous research, prolonged school closing, combined with home confinement, can have negative effects on children’s physical and psychological health [2,3]. Refs. [46,47] During the total lockdown, the opportunities of movement like PA classes, organized PA or any type of free play are restricted [48]. That is why a recent study reported a decrease of 435 min/week of physical activity and 2.3 h/week of sport activities in Chinese children [38].

Several previous studies that explored the consequences of periods without school, such as weekends and summer vacations, showed weight gain linked to a decrease in PA, irregular sleep schedules and increase in time of digital media use [49,50].

In the context of the novel COVID-19 outbreak, it is worth noting that PA allows reducing the risk for developing inflammation, excess body mass and non-communicable diseases known to compromise immune function [51]. The immune system is sensitive to PA depending on the intensity and duration of effort and type of exercise [52]. For prevention, the World Health Organization supported individuals to organize a regular engagement in PA to maintain mental and physical health even during home confinement [1,53].

Thus, it is better to replace ST with active video games or online classes of PA [54] while reducing the ST before sleep. In fact, active video games are currently used in therapeutic education, prevention, treatment of various medical conditions, rehabilitation, and health care or as a means to fight sedentariness [55].

Furthermore, recent large survey study suggested that low PA during COVID-19 home confinement [56] was associated with poorer sleep quality in the general population [57,58]. The present results confirm this suggestion in children and showed that (i) global score of PA was negatively correlated with “daytime dysfunction” and (ii) reduced PA activities in both genders were accompanied with higher PSQI score. Therefore, it is recommended to increase PA and to reduce sedentary behaviors during home confinement in order to improve sleep quality in children.

## 5. Limitations

This is the first study to investigate the effect of COVID19 home confinement on lifestyle behaviors of Tunisian children with a specific focus on possible gender effect. However, there are some limitations that need to be considered. First, we did not use any objective measurement for the evaluation of sleep quality and PA levels. Indeed, smart watches or other wearable devices were not available to all participants to allow continuous assessment of sleep and PA. Second, it would be more advantageous if we investigated larger population. Third, possible limitations could be related to the use of the cross-sectional design assessing the “before” home confinement condition retrospectively. However, it should be noted that home confinement was a sudden measure. Therefore, researchers worldwide were not able to develop and spread their survey at “before” home confinement. Finally, it should be also noted that self-reporting, confirmation bias, as well as having a parent assist with the child’s reporting, may also have affected the results. Indeed, the authors of the present study expect that confirmation bias might bias the PA’s results upwards (overestimating the true effect), while a response bias resulting from parental assistance would likely have biased the results of ST downwards and might have resulted in underestimating the home confinement effect. Therefore, the present findings should be interpreted with caution.

## 6. Conclusions

In this cross-sectional study, an increase in the use of digital media and a decrease in the frequency of PA were observed in Tunisian children. These lifestyle changes were accompanied with disturbances in their sleep–wake rhythms. Importantly, these negative effects of COVID-19 home confinement were more pronounced in girls compared to boys. In order to recover these unwanted consequences, it is advisable to maintain an adequate sleep rhythm and 60 min of moderate to vigorous daily PA [59] while reducing exposition to digital devices. Importantly, special care should be given to maintain these daily behaviors in girls during home confinement.

## Figures and Tables

**Table 1:** Characteristics of parents.

Education of Father		Education of Mother		Profession of Father		Profession of Mother	
Level	Percentages	Level	Percentages	Type	Percentages	Type	Percentages
Illiterate	0%	Illiterate	0%	Unemployed	4%	Housewife	21%
Primary school	18%	Primary school	16%	Public sector employed	31%	Public sector employed	31%
Secondary school	32%	Secondary school	2%	Private sector employed	37%	Private sector employed	27%
Higher school	38%	Higher school	42%	Self employed	28%	Self employed	16%
Master/doctorate degree	12%	Master/doctorate degree	14%	Student	0%	Student	5%

**Table 2:** Reponses to the PSQI recorded before and during home confinement.

**Variable**	**BOYS**				**GIRLS**				**Whole Sample**				**ANOVA**			**Δ_before =_ G_before_-B_before_**	**Δ_during =_ G_during_-B_during_**
	**Before**	**During**	**Δ_boys =_ D_boys_-B_boys_**	**% of Change**	**Before**	**During**	**Δ_girls =_ D_girls_-B_girls_**	**% of Change**	**Before**	**During**	**Δ_girls =_ D_girls_-B_girls_**	**% of Change**	**Confinement**	**Gender**	**Interaction**		
**PSQI**																	
Subjective sleep quality (AU1)	0.37 ± 0.52	0.98 ± 0.61	0.61 ***	+41.67	0.6 ± 0.61	1.25 ± 0.6	0.65 ***	+25	0.48 ± 0.57	1.11 ± 0.61	0.63 ***	+66.67	F = 89.88 *p* < 0.001 ηp^2^ = 0.47	F = 6.88 *p* < 0.01 ηp^2^ = 0.06	*p* = 0.81	0.09 ^$^	0.27 ^$^
Sleep latency (min)	11.23 ± 7.85	16.73 ± 8.69	5.5 ***	+119.6	13.25 ± 6.74	19.65 ± 8.02	6.4 ***	+114.9	12.2 ± 7.37	18.13 ± 8.46	5.93 ***	+205	F = 42.89 *p* < 0.001 ηp^2^ = 0.3	*p* = 0.05	*p* = 0.62	2.02	2.92
Sleep duration (hour)	8.78 ± 0.95	8.71 ± 0.93	−0.07	−0.01	8.65 ± 0.72	8.73 ± 0.78	0.08	+1.46	8.72 ± 0.85	8.73 ± 0.87	0.001	+4.63	*p* = 0.97	*p = 0.64*	*p* = 0.45	−0.13	−0.05
Habitual sleep efficiency (%)	92.23 ± 4.75	87.63 ± 5.54	−4.6 ***	−4.45	91.71 ± 4.4	88.83 ± 4.99	−2.88 **	−3.03	91.7 ± 4.69	88.12 ± 5.37	−3.58 ***	−2.29	F = 39.66 *p* < 0.001 ηp^2^ = 0.28	*p* = 0.54	*p* = 0.24	−0.52	1.2
Sleep disturbances (AU1)	0.48 ± 0.57	1.03 ± 0.55	0.55 ***	+26.09	0.67 ± 0.47	1.29 ± 0.54	0.62 ***	+34.38	0.57 ± 0.53	1.16 ± 0.56	0.59 ***	+66.67	F = 94.83 *p* < 0.001 ηp^2^ = 0.49	F = 5.97 *p* = 0.016 ηp^2^ = 0.05	*p* = 0.58	0.19	0.26 ^$^
Daytime dysfunction (AU1)	0.59 ± 0.56	1.34 ± 0.48	0.75 ***	+46.55	0.63 ± 0.56	1.6 ± 0.57	0.97 ***	+50	0.61 ± 0.56	1.47 ± 0.54	0.85 ***	+66.67	F = 150.09 *p* < 0.001 ηp^2^ = 0.6	*P* = 0.09	*p* = 0.1	0.04	0.26 ^$^
Global PSQI score (AU2)	1.94 ± 1.62	4.59 ± 2.12	2.65 **	173.44	2.33 ± 1.09	5.73 ± 1.62	3.4 ***	+175.19	2.13 ± 1.4	5.15 ± 1.9	3.02 ***	+82.22	F = 320.58 *p* < 0.001 ηp^2^ = 0.76	F = 6.96 *p* = 0.01 ηp^2^ = 0.066	*p* = 0.03	0.39	1.14 ^$^

AU1: Arbitrary Unit (from 0 to 3); AU2: Arbitrary Unit (from 0 to 21), **, ***: significant difference between before-during confinement (*p* < 0.01 and *p* < 0.001, respectively); ^$^: significant difference between girls and boys at *p* < 0.05.

**Table 3:** Responses to daily screen time questionnaire recorded before and during home confinement.

**Variable**	**BOYS**				**GIRLS**				**Whole Sample**				**ANOVA**			**Δ_before =_ G_before_-B_before_**	**Δ_during =_ G_during_-B_during_**
	**Before**	**During**	**Δ_boys =_ D_boys_-B_boys_**	**% of Change**	**Before**	**During**	**Δ_girls =_ D_girls_-B_girls_**	**% of Change**	**Before**	**During**	**Δ_population =_ D_pop_-B_pop_**	**% of Change**	**Confinement**	**Gender**	**Interaction**		
**Screen time**																	
Diurnal (Hour/day)	1.11 ± 0.58	3.15 ± 0.99	2.04 ***	+164.13	1.38 ± 0.67	3.63 ± 0.1.0	2.25 ***	+148.8	1.24 ± 0.63	3.38 ± 1.06	2.14 ***	+150	F = 529.72 *p* < 0.001 ηp^2^ = 0.84	F = 6.29 *p* = 0.013 ηp^2^ = 0.06	*p* = 0.25	0.27 ^$^	0.48 ^$^
Nocturnal (Hour/day)	0.21 ± 0.37	0.72 ± 0.75	0.51 ***	+116.66	0.34 ± 0.47	0.94 ± 0.43	0.6 ***	+82.02	0.23 ± 0.42	0.76 ± 0.69	0.53 ***	+93.33	F = 51.29 *p* < 0.001 ηp^2^ = 0.53	F = 8.95 *p* < 0.001 ηp^2^ = 0.08	*p* = 0.2	0.13 ^$^	0.22 ^$^
Global (Hour/day)	1.32 ± 0.69	4.08 ± 1.34	2.76 ***	+189.13	1.72 ± 0.82	4.91 ± 1.22	3.19 ***	+176.36	1.53 ± 0.79	4.45 ± 1.41	2.92 ***	+111.11	F = 619.51 *p* < 0.001 ηp^2^ = 0.86	F = 11.14 *p* < 0.001 ηp^2^ = 0.1	F = 5.54 *p* = 0.02 ηp^2^ = 0.05	0.4 ^$^	0.83 ^$^

***: significant difference between before-during confinement (*p* < 0.001) ^$^: significant difference between girls and boys at *p* < 0.05.

**Table 4:** Responses to level of PA before and during confinement.

**Variable**	**BOYS**				**GIRLS**				**Whole Sample**				**ANOVA**			**Δ_before =_ G_before_-B_before_**	**Δ_during =_ G_during_-B_during_**
	**Before**	**During**	**Δ_boys =_ D_boys_-B_boys_**	**% of Change**	**Before**	**During**	**Δ_girls =_ D_girls_-B_girls_**	**% of Change**	**Before**	**During**	**Δ_population =_ D_pop_-B_pop_**	**% of Change**	**Confinement**	**Gender**	**Interaction**		
**Physical Activity**																	
Sedentary behavior	1.09 ± 0.29	2.28 ± 1.03	1.19 ***	+115.38	1.02 ± 0.14	2.29 ± 1.02	1.27 ***	+126.67	1.06 ± 0.24	2.29 ± 1.03	1.23 ***	+133.33	F = 134.6 *p* < 0.001 ηp^2^ = 0.58	*p* = 0.74	*p* = 0.72	−0.7	0.01
Leisure PA	8.36 ± 4.29	5.38 ± 4.43	−2.98 **	−35.56	8.17 ± 4.36	5.26 ± 4.47	−2.91 **	−35.31	8.27 ± 4.32	5.32 ± 4.45	−2.95 ***	−39.09	F = 26.15 *p* < 0.001 ηp^2^ = 0.21	*p* = 0.81	*p* = 0.95	−0.18	−0.12
Daily PA	7.17 ± 2.24	5.4 ± 2.01	−1.77 ***	−16.06	7.48 ± 2.35	5.13 ± 1.74	−2.35 ***	−26.94	7.32 ± 2.3	5.27 ± 1.89	−2.05 ***	−33.33	F = 62.61 *p* < 0.001 ηp^2^ = 0.39	*p* = 0.96	*p* = 0.26	0.31	−0.27
Total PA score	16.63 ± 5.44	13.07 ± 6.15	−0.56 **	−7.05	16.05 ± 5.07	12.42 ± 5.16	−3.63 ***	−17.26	16.57 ± 5.26	12.76 ± 5.7	−3.81 ***	−28.06	F = 29.86 *p* < 0.001 ηp^2^ = 0.23	*p* = 0.64	*p* = 0.7	−0.58	−0.65

**, ***: significant difference between before-during confinement (*p* < 0.01 and *p* < 0.001, respectively).

## Data Availability

Data are available from the authors (R.A., or A.A.) upon reason-able request.

## Supplementary Materials

The following are available online at https://www.mdpi.com/1660-4601/18/6/3065/s1, Table S1: Reponses to the PSQI before and during confinement. Table S2: Responses to daily screen time questionnaire before and during confinement. Table S3: Responses to the level of PA before and during confinement.
