# Supplementary material for: Effect of COVID-19-Related Home Confinement on Sleep Quality, Screen Time and Physical Activity in Tunisian Boys and Girls: A Survey
Source: Int J Environ Res Public Health. 2021 Mar 16;18(6):3065. doi: 10.3390/ijerph18063065 (PMC8002304; doi:10.3390/ijerph18063065)
Supplement: Supplementary file 1 [file ijerph-18-03065-s001.pdf]

Table S1. Reponses to the PSQI before and during confinement.

Variable	BOYS				GIRLS				All population			
	Before		During		Before		During		Before		During	
	Mean± SD	Median [max-min]	Mean± SD	Median [max-min]	Mean± SD	Median [max-min]	Mean± SD	Median [max-min]	Mean± SD	Median [max-min]	Mean± SD	Median [max-min]
PSQI												
Subjective sleep quality (AU)	0.37± 0.52	0 [0-2]	0.98± 0.61	1 [0-2]	0.6± 0.61	1 [0-2]	1.25± 0.6	1 [0-2]	0.48± 0.57	0 [0-2]	1.11± 0.61	1 [0-2]
Sleep latency (min)	11.23± 7.85	10 [0-35]	16.73± 8.69	20 [5-35]	13.25± 6.74	15 [2-25]	19.65± 8.025	20 [2-35]	12.2± 7.37	10 [0-35]	18.13± 8.46	20 [2-35]
Sleep duration (hour)	8.78± 0.95	9 [7-11]	8.71± 0.93	8 [7-11]	8.65± 0.72	9 [8-10]	8.73± 0.78	8 [8-10]	8.72± 0.85	9 [7-11]	8.73± 0.87	8 [7-11]
Habitual sleep efficiency (%)	92.23± 4.75	94 [80-100]	87.63± 5.54	88 [80-99]	91.71± 4.4	90 [83-99]	88.83± 4.99	88 [80-99]	91.7± 4.69	94 [80-100]	88.12± 5.37	88 [80-99]
Sleep disturbances (AU)	0.48± 0.57	0 [0-2]	1.03± 0.55	1 [0-2]	0.67± 0.47	1 [0-1]	1.29± 0.54	1 [0-2]	0.57± 0.53	1 [0-2]	1.16± 0.56	1 [0-2]
Daytime dysfunction (AU)	0.59± 0.56	1 [0-2]	1.34± 0.48	1 [1-2]	0.63± 0.56	1 [0-2]	1.6± 0.57	2 [0-2]	0.61± 0.56	1 [0-2]	1.47± 0.54	1 [0-2]
Global PSQI score (AU)	1.94± 1.62	1.5 [0-6]	4.59± 2.12	5 [1-9]	2.33± 1.09	2 [0-5]	5.73± 1.62	6 [2-9]	2.13± 1.4	2 [0-6]	5.15± 1.9	5 [1-9]

Table S2. Responses to daily screen time questionnaire before and during confinement.

Variable	BOYS				GIRLS				All population			
	Before		During		Before		During		Before		During	
	Mean± SD	Median [max-min]	Mean± SD	Median [max-min]	Mean± SD	Median [max-min]	Mean± SD	Median [max-min]	Mean± SD	Median [max-min]	Mean± SD	Median [max-min]
Screen time												
Diurnal (Hour/day)	1.11± 0.58	1 [0-2]	3.15± 0.99	3 [1-6]	1.38± 0.67	1 [0-2]	3.63± 0.7	4 [1-6]	1.24± 0.63	1 [0-2]	3.38± 1.06	3 [1-6]
Nocturnal (Hour/day)	0.21± 0.37	0 [0-1]	0.72± 0.75	0.5 [0-5]	0.34± 0.47	0 [0-1]	0.94± 0.43	1 [0-2]	0.23± 0.42	0 [0-1]	0.76± 0.69	1 [0-5]
Global (Hour/day)	1.32± 0.69	1 [0-3]	4.08± 1.34	4 [2-9]	1.72± 0.82	2 [0-3]	4.91± 1.22	5 [2-8]	1.53± 0.79	2 [0-3]	4.45± 1.41	4 [2-9]

Table S3. Responses to the level of PA before and during confinement.

Variable	BOYS				GIRLS				All population			
	Before		During		Before		During		Before		During	
	Mean± SD	Median [max-min]	Mean± SD	Median [max-min]	Mean± SD	Median [max-min]	Mean± SD	Median [max-min]	Mean± SD	Median [max-min]	Mean± SD	Median [max-min]
Physical activity												
Sedentary behavior	1.09± 0.29	1 [1-2]	2.28± 1.03	2 [1-5]	1.02± 0.14	1 [1-2]	2.29± 1.02	2 [1-5]	1.06± 0.24	1 [1-2]	2.29± 1.03	2 [1-5]
Leisure PA	8.36± 4.29	10 [1-14]	5.38± 4.43	6.5 [1-14]	8.17± 4.36	10 [1-14]	5.26± 4.47	2 [1-14]	8.27± 4.32	10 [1-14]	5.32± 4.45	5 [1-14]
Daily PA	7.17± 2.24	7 [4-12]	5.4± 2.01	5 [1-10]	7.48 ± 2.35	7 [4-14]	5.13± 1.74	4 [1-10]	7.32± 2.3	7 [4-14]	5.27± 1.89	5 [1-10]
Total PA score	16.63± 5.44	18 [6-21]	13.07± 6.15	13 [6-27]	16.05± 5.07	18 [6-25]	12.42± 5.16	11 [6-25]	16.57± 5.26	18 [6-25]	12.76± 5.7	12 [6-27]
